# Treatment-Resistant Chromoblastomycosis Successfully Managed With Surgical Excision

**DOI:** 10.7759/cureus.73619

**Published:** 2024-11-13

**Authors:** Matthew B Martinelli, Clay J Cockerell, Philip R Cohen

**Affiliations:** 1 Natural Science, Highland Park High School, Dallas, USA; 2 Dermatology, Texas Skin Surgery Center, Plano, USA; 3 Dermatopathology, Cockerell Dermatopathology, Dallas, USA; 4 Dermatology, University of California Davis Medical Center, Sacramento, USA; 5 Dermatology, Touro College of Osteopathic Medicine, Vallejo, USA

**Keywords:** bodies, chromoblastomycosis, copper pennies, dematiaceous, excision, fungus, itraconazole, medlar, muriform, terbinafine

## Abstract

Chromoblastomycosis is an uncommon, chronic granulomatous fungal infection of the skin and subcutaneous tissue. Chromoblastomycosis is most commonly caused by the traumatic inoculation of dematiaceous (pigmented) fungi, most commonly *Fonsecaea *species, *Phialophora *species, and *Cladophialophora *species. Chromoblastomycosis usually affects agricultural workers in tropical and subtropical climates. The World Health Organization classifies chromoblastomycosis as a neglected tropical and occupational disease that commonly affects middle-aged men in poor to middle-income countries. The cutaneous lesions of chromoblastomycosis typically affect the lower extremities and present as polymorphous, hyperkeratotic, or fungating small papules, plaques, verrucous nodules, or ulcers; therefore, a high degree of clinical suspicion is necessary to consider the diagnosis of chromoblastomycosis. The diagnosis is made by visualization of the thick-walled pigmented structures referred to as sclerotic bodies (also known as Medlar bodies or muriform bodies) or pigmented septate hyphae or both on a biopsy specimen of the lesion. Treatment may consist of locally destructive techniques, prolonged systemic antifungal therapy, and/or surgical excision. In this paper, we present an immunocompetent 80-year-old Caucasian woman who developed an isolated lesion of chromoblastomycosis on the forearm while gardening in Texas, a non-endemic area for the disease. Her infection was refractory to systemic antifungal medications and cryotherapy with liquid nitrogen. Ultimately, her fungal infection was successfully treated with a wide local surgical excision of the infectious cutaneous lesion.

## Introduction

Chromoblastomycosis was first reported in Brazil by Max Rudolph and was first described histologically by Lane and Medlar in 1915 [1,2]. The World Health Organization defines a neglected tropical disease to be an endemic disease primarily affecting low-income populations [3]. The World Health Organization has categorized chromoblastomycosis as a neglected tropical disease [3].

Chromoblastomycosis is generally found in endemic tropical and subtropical regions such as Asia, Africa, Latin America, and the Caribbean [4-7]. The highest prevalence rates are reported in South America, followed by Africa [7]. Chromoblastomycosis is a chronic, granulomatous mycosis of the skin and subcutaneous tissue caused by the percutaneous inoculation of various pigmented fungi typically present in soil, plants, and decomposing wood within affected endemic regions [3]. The most common causative organisms are *Fonsecaea pedrosoi*,* Fonsecaea compactum*, *Phialophora verrucosa*, and *Cladophialophora carrionii* [1]. 

The cutaneous manifestations of chromoblastomycosis are varied. Infection-associated lesions are pleomorphic and can consist of papules and nodules, plaques, verrucous lesions, scar-like plaques, or ulcers; the lesions most commonly occur on the lower extremities [8]. Systemic antifungal therapy is typically the first-line treatment; however, locally destructive techniques and/or surgical excision may be considered for refractory cases. 

We report a case of an 80-year-old woman in Texas, a non-endemic region, who developed a solitary lesion of chromoblastomycosis on her right forearm. Her fungal skin lesion was initially refractory to oral antifungal therapy and cryotherapy. The woman’s chromoblastomycosis infection was subsequently successfully treated with surgical excision.

## Case presentation

An 80-year-old Caucasian woman presented for the evaluation and treatment of a non-resolving lesion on her right forearm present for approximately five months. Her past medical history was significant for hypertension controlled with metoprolol, as well as hypercholesterolemia treated with rosuvastatin. The patient reported a history of chronic heart failure with reduced ejection fraction treated with oral sacubitril/valsartan. She also had a history of coronary artery disease and was anticoagulated with apixaban and 81 milligrams of aspirin each day. Her medical history was unremarkable for immunosuppression. She was a non-smoker and a retired medical professional.

**Figure 1:**
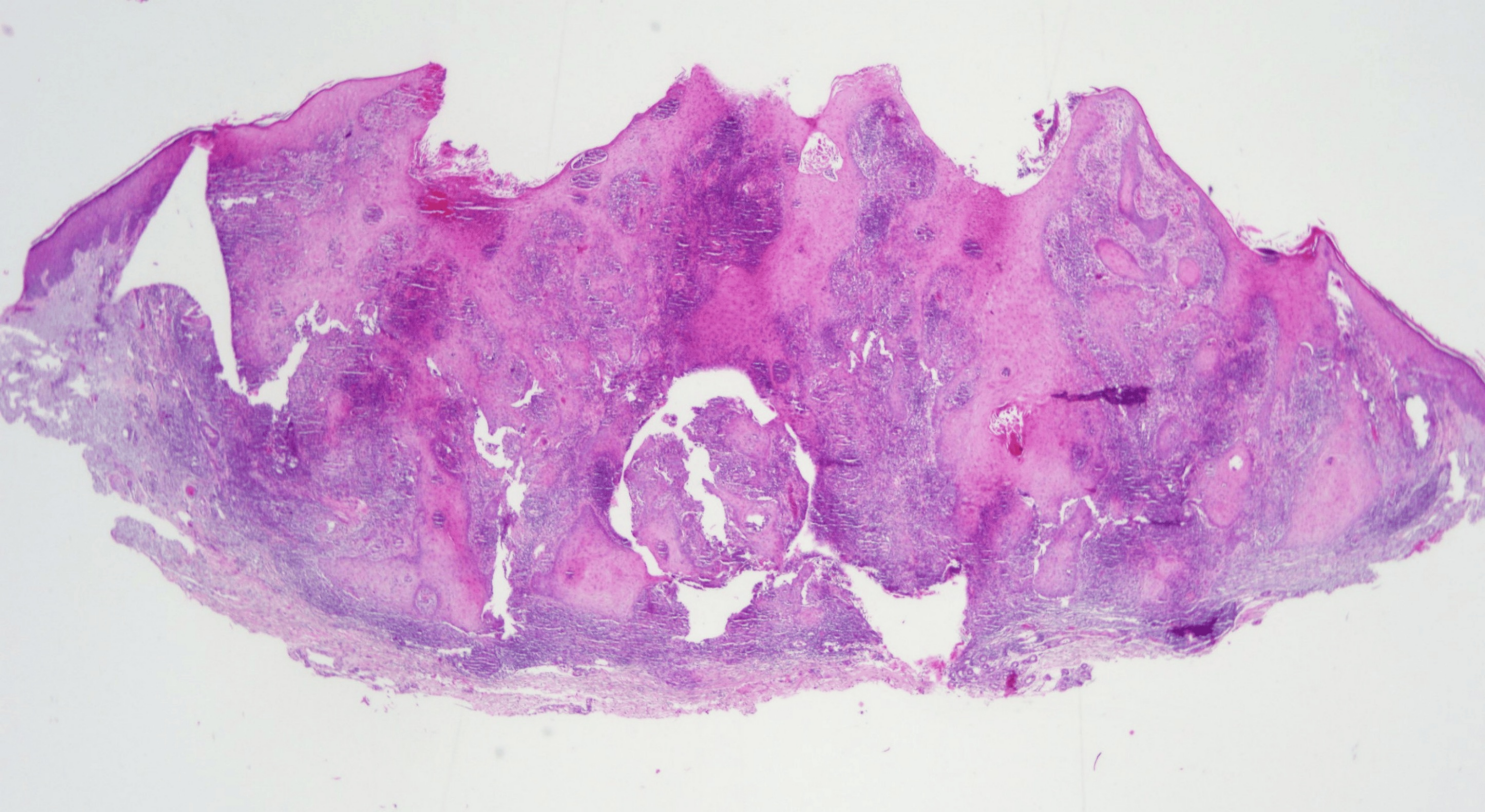
Microscopic examination of a shave biopsy of chromoblastomycosis A low magnification view of the specimen demonstrates epidermal hyperplasia and a suppurative granulomatous dermal infiltrate (Hematoxylin and eosin, x 2).

The patient stated that the lesion developed at the site of a wound she received while gardening in her yard. She is an avid gardener and recalls getting stuck by a thorn or splinter that broke the skin. She presented to her dermatologist where an initial impression of squamous cell carcinoma was made, and a shave biopsy of the lesion was performed. Microscopic examination of the tissue specimen revealed epidermal hyperplasia and a suppurative granulomatous infiltrate (Figure 1).

**Figure 2:**
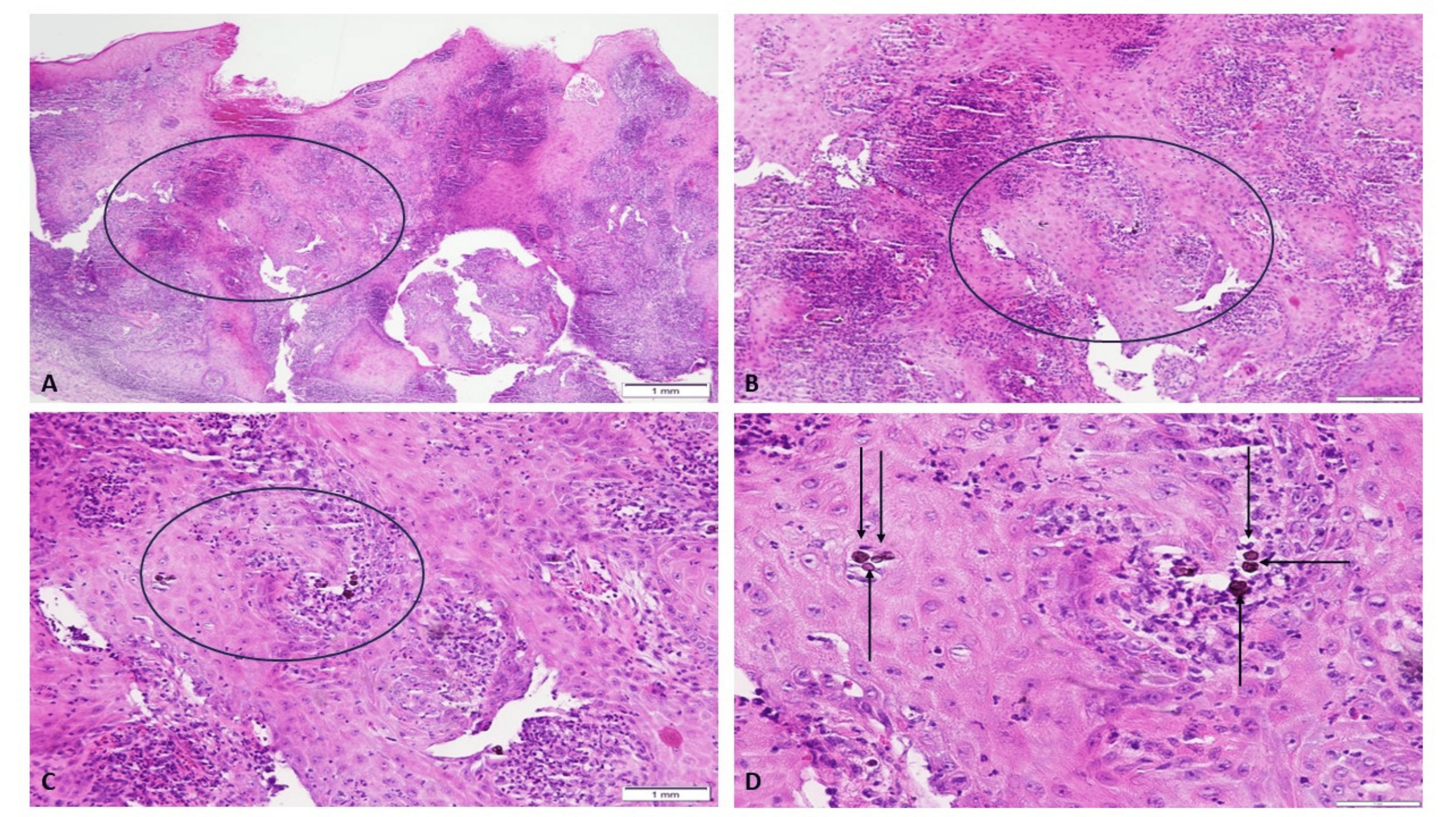
Pathologic changes observed at higher magnification of the shave biopsy specimen of chromoblastomycosis Low magnification (A) and higher magnification (B-D) views of the granulomatous infiltrate and the presence of multiple thick-walled sclerotic (muriform or Medlar) bodies. The black circles in images A, B, and C include the portion of the dermal infiltrate that is shown in the subsequent photomicrograph. The black arrows in image D point to the brown-staining sclerotic bodies (Hematoxylin and eosin: A: x4, B: x10, C: x20, D: x40).

Higher magnification views of the infiltrate revealed the presence of numerous brown, thick-walled sclerotic (Medlar) bodies (Figure 2). Based on these characteristic histopathologic findings, a diagnosis of chromoblastomycosis was made.

The woman was initially placed on a course of itraconazole but was forced to discontinue this after several weeks due to the development of cardiac Q-T prolongation caused by this medication. She underwent three treatments of liquid nitrogen cryotherapy with little to no improvement at the site. Terbinafine 250 milligrams daily was then initiated; after three months, there was no resolution or reduction in the size of the lesion. Given the refractory nature of the lesion, she was referred to our office to discuss surgical management of her fungal infection.

**Figure 3:**
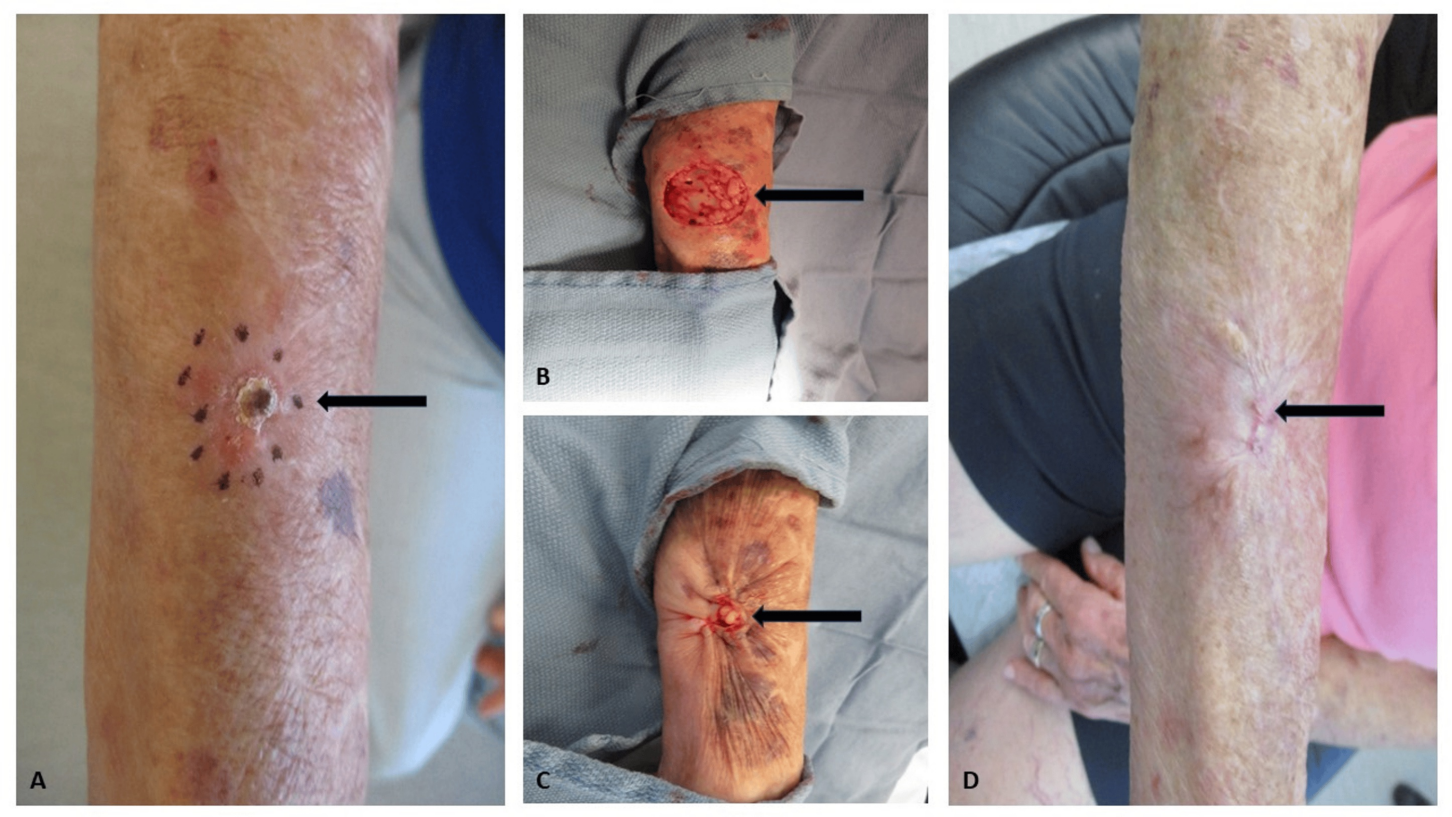
Clinical presentation and treatment photos of chromoblastomycosis in an 80-year-old Caucasian woman The residual fungal infection (A) presented as an isolated 1.5 x 1.5 centimeter erythematous keratotic plaque with hemorrhagic crust on the right ventral forearm; a black arrow points to the lesion, which is surrounded by purple dots. The black arrow points to the surgical wound (B) immediately following a wide local excision with 0.5 centimeter peripheral margins to muscular fascia. A black arrow points to the wound site after a purse-string repair of the defect (C). A black arrow points to the healed surgical scar at 3-month follow-up (D); there are no clinical signs of residual fungal disease.

Cutaneous examination of the right ventral forearm revealed a 1.5x1.5 centimeter erythematous keratotic plaque with central hemorrhagic crust (Figure 3). There were no other lesions present, and there was no ipsilateral epitrochlear or axillary lymphadenopathy palpated. The patient was afebrile, with no evidence of distress, and had normal vital signs. 

The patient wanted to avoid another invasive diagnostic procedure. Therefore, a repeat biopsy for tissue culture was not performed. As the lesion remained refractory to several months of systemic antifungal therapy and locally destructive techniques, she opted for excision of the lesion.

A wide local excision with 0.5-centimeter margins of normal-appearing skin was performed using local anesthesia (Figure 3). The resulting wound measured approximately 2.5x2.5 centimeters and extended to the depth of the muscular fascia. Considering the atrophic nature of her skin and her anticoagulated status, and in an effort to preserve proper margin orientation pending pathology results, a cuticular purse-string repair was elected in lieu of a more extensive primary closure. The excised specimen was sent to pathology, and all margins of excision were confirmed clear of fungal organisms. At her three-month follow-up visit, the site was well-healed and without any clinical evidence of recurrent fungal disease. She will continue regular follow-up examinations every six months to assess for signs of clinically recurrent fungal disease.

## Discussion

Chromoblastomycosis is a chronic, progressive, granulomatous fungal infection of the skin and subcutaneous tissue caused by dematiaceous fungi, primarily of the genera Fonsecaea, Phialophora, and Cladophialophora [4,9,10]. The disease is endemic to tropical and subtropical regions of Latin America, Africa, Asia, and the Caribbean and is rarely seen in the United States.

The fungus is found in soil, plants, and decaying wood. Pathogenesis typically occurs via traumatic percutaneous inoculation of the organism via contact with splinters, thorns, or decaying plants [11]. Notably, the patient reported gave a history of being stuck by thorns or splinters while working in her garden that preceded the appearance of her forearm lesion.

Once inoculated in the skin, chromoblastomycosis is a locally progressive disease that can follow a chronic course with continued growth for many years. Chromoblastomycosis commonly occurs in immunocompetent hosts, such as the patients described in this report. However, immunosuppressed solid organ transplant recipients and patients with HIV infection may be at higher risk of infection [8,10]. Additionally, patients taking oral tacrolimus may experience more frequent, severe, and treatment-resistant infections [8].

Typically, chromoblastomycosis lesions are present on the lower extremities of middle-aged men working in rural tropical endemic areas. The classic initial lesion is often an asymptomatic or mildly pruritic red to violaceous papule. The initial lesion may remain circumscribed to the inoculation site for months or even years, before expanding and growing centrifugally. The clinical differential diagnosis of all early stage lesions includes tumors such as squamous cell carcinoma, foreign body reaction, or other deep fungal infection or an atypical mycobacterial infection [3].

The lesions of chromoblastomycosis are polymorphous and may present a diagnostic challenge [1]. More advanced chromoblastomycosis fungal lesions can appear annular with central scarring; they can also present as verrucous and vegetative plaques, often affecting an entire limb [1,3,5,6]. Rare reported presentations include an isolated dome-shaped papule on a finger, an enlarging erythematous nodule on the jawline, a mycetoma-like lesion of the dorsal foot, discrete and confluent infiltrated papules and pustules of a forearm, and nodulo-ulcerative lesions of the legs [8,12-15]. Long-standing chronic lesions may be complicated by secondary bacterial infections, lymphedema, ankylosis, hematogenous dissemination, and malignant transformation, typically squamous cell carcinoma [3,12].

The diagnosis of chromoblastomycosis can be confirmed by histopathological examination of a skin biopsy specimen, which demonstrates the characteristic-appearing muriform pigmented fungal cells (also referred to as Medlar bodies or copper pennies) within the granulomatous infiltrates in the dermis [1,3]. If needed, fungal cultures may also be used to identify the causative organism [1]. Given our patient’s history of exposure and injury, as well as the characteristic muriform cells seen in her biopsy specimen, additional cultures were not needed to confirm her diagnosis.

Oral antifungal therapy is the mainstay of treatment for chromoblastomycosis. Long-term itraconazole therapy at doses of 200-400 milligrams daily, generally given for six to 12 months depending on clinical severity, is first-line therapy [2,3,5]. Pulsed dosing of itraconazole (400 milligrams per day for seven days per month) has also been reported to be effective and may also offer better cost efficiency with improved compliance [2,3,5]. Terbinafine is a second-line treatment agent and demonstrates antifungal activity* in vitro* against the etiologic agents of chromoblastomycosis; recommended dosing is 250-500 milligrams daily [5]. Finally, posaconazole at doses of 800 milligrams daily may be considered for refractory cases, but it remains cost-prohibitive for many patients [2].

Locally destructive techniques can be used in conjunction with systemic antifungals. These may be employed for small or localized lesions. Heat therapy, in which local heat is used to produce surface temperatures of 42-46 degrees Centigrade, has been shown to be effective at reducing fungal growth with minimal adverse events; however, daily treatment sessions of several hours for two to six months are required for best results [3].

Cryosurgery via the application of liquid nitrogen to affected skin may also be used in conjunction with antifungals in limited disease. However, its use is limited due to risks of scarring and hypopigmentation, and freezing time and depth have not been standardized [3,16]. This approach to treatment was unsuccessful in the woman described in this report.

Surgical excision may also be considered for localized disease or lesions refractory to antifungal therapy. Wide local surgical excision, as well as Mohs micrographic surgery, have been reported to be successful in the proper clinical context [11,17]. Additionally, there are reports of chromoblastomycosis being successfully treated by oral terbinafine to reduce lesion size, followed by surgical excision of the remaining disease [18].

The chromoblastomycosis infection in the patient reported in this paper had no response after several months of terbinafine treatment. Considering the localized nature of her disease involvement, a wide local excision was employed with success. The wound was successfully partially closed using a cuticular purse-string suture and subsequently completely healed without any clinical evidence of residual fungal infection [19].

## Conclusions

An elderly immunocompetent woman with chromoblastomycosis on her upper extremity has been reported. This atypical clinical presentation in a non-endemic area, which has a low prevalence for this disease, highlights the importance of considering chromoblastomycosis in the clinical differential diagnosis of isolated crusted or verrucous lesions. Despite its localized and limited nature, our patient’s chromoblastomycosis infection was nevertheless refractory to oral antifungal therapy and local cryotherapy; it required surgical excision for complete extirpation and resolution of the fungal infection.
